# Digital Order System Redesign in Emergency-to–Intensive Care Unit Admissions: Prospective Observational Study

**DOI:** 10.2196/88230

**Published:** 2026-10-09

**Authors:** Ding-Kuo Chien, Wei Hung Chang, Chih Wei Ten, Jung-Mei Tsai, Li-Kuo Kuo, Shih-Yi Lee

**Affiliations:** 1 Department of Emergency Medicine, MacKay Memorial Hospital, No. 92, Sec. 2, Zhongshan North Road., Taipei, 104217, Taiwan, 886 0975835137; 2 Department of Medicine, MacKay Medical University, New Taipei City, Taiwan; 3 Department of Critical Care Medicine, MacKay Memorial Hospital, No. 92, Sec. 2, Zhongshan North Road, Taipei, 104217, Taiwan, 886 0975835137; 4 Department of Nursing, MacKay Memorial Hospital, Taipei, Taiwan; 5 Center for Evidence-Based Medicine, MacKay Memorial Hospital, Taipei, Taiwan; 6 MacKay Junior College of Medicine, Nursing, and Management, Taipei, Taiwan

**Keywords:** emergency ICU, digital order system, workflow redesign, informatics, traceability, intensive care unit, ICU

## Abstract

**Background:**

Emergency-to–intensive care unit (ICU) admissions are high-stakes transitions in care, where delays or documentation errors can compromise patient safety and disrupt operational efficiency. Although digital order systems can support these workflows, traditional platforms often lack real-time traceability and structured input logic, leaving them vulnerable to duplicate submissions, lost orders, and misaligned ICU bed reservations.

**Objective:**

The study aimed to examine whether an updated digital order system was associated with differences in emergency-to-ICU admission workflows, including order validity, documentation-error frequencies, and ICU reservation alignment, compared with those of the traditional platform.

**Methods:**

We conducted a single-center retrospective historical-control formative evaluation of emergency-to-ICU admissions processed through traditional (2023) and updated (2024) digital order systems at a tertiary medical center. Prospectively generated system log and ICU-reservation data were analyzed to quantify order validity, error subtypes (duplicate, lost, and tracking), and admission-to-reservation alignment. Proportions were compared using chi-square tests, with risk ratios (RRs) and absolute differences in proportions reported. Statistical significance was defined as *P*<.05.

**Results:**

The updated system was associated with higher admission-order validity (54.1%-75.4%; RR 1.39, 95% CI 1.25‐1.54; *P*<.001) and lower erroneous-order frequency (45.9%-24.6%; RR 0.54, 95% CI 0.44‐0.66; *P*<.001). Duplicate and lost orders declined, whereas tracking-error frequency showed no meaningful change. ICU reservation alignment also differed, with the admission-to-reservation ratio rising from 0.68 to 0.78 (RR 0.88, 95% CI 0.77-0.99; *P*=.03).

**Conclusions:**

The updated digital order system was associated with more consistent emergency-to-ICU workflow patterns, including higher order validity, fewer documentation errors, and closer alignment between admission intent and ICU reservations. These findings are consistent with literature on structured electronic order systems, digital-workflow standardization, and timestamp integrity, and they highlight the potential value of structured, timestamp-driven digital infrastructures in supporting more reliable high-acuity workflows. Further evaluation using time-motion analysis, interaction-log metrics, or mixed methods assessment is warranted to examine the robustness and generalizability of these patterns.

## Introduction

Timely and accurate intensive care unit (ICU) admissions from the emergency department (ED) are essential for optimizing patient outcomes and hospital resource usage. Delays in ICU transfers have been consistently associated with increased mortality, prolonged hospitalization, and elevated health care costs [1-4]. In high-acuity environments, effective coordination of clinical decisions, documentation, and bed logistics is required to maintain both clinical safety and operational efficiency.

The digitization of hospital workflows has transformed emergency care delivery, replacing paper-based processes with structured digital platforms that support real-time documentation and cross-team communication. Despite these advances, traditional digital systems often underperform in dynamic emergency settings. They may lack integration across departments, provide limited real-time status visibility, or rely on manual reconciliation steps that introduce fragmentation and reduce traceability [5-7]. These limitations can lead to erroneous admission orders, documentation gaps, and inefficient resource allocation—issues that are particularly consequential during ED-initiated ICU transitions.

Recent work in smart-hospital infrastructure has introduced increasingly sophisticated tools for synchronizing clinical orders, documentation, and bed-management processes. Digital coordination centers have demonstrated measurable improvements in cross-department communication and operational visibility, enabling hospitals to streamline patient flow and reduce coordination failures [5]. Parallel advances in intelligent-ICU design highlight the role of integrated sensing, data fusion, and real-time monitoring in supporting high-acuity decision-making and workflow reliability [6]. Broader digital-transformation frameworks further show that big-data–enabled systems can enhance process efficiency, information integration, and organizational responsiveness in complex health care environments [6]. Emerging technologies such as hands-free documentation tools [7], Internet of Things–based smart-hospital architectures [8], and digital emergency-response platforms [9] collectively demonstrate that modern clinical environments increasingly depend on accurate, real-time digital information to support coordination and traceability.

Despite these advances, most existing research focuses on clinical outcomes, such as the impact of delayed ICU admission on mortality [1] or on high-level digital-infrastructure design, rather than on the validity and traceability of admission-order workflows themselves. Evidence specific to ED-initiated ICU admission processes remains limited. Few studies have examined how digital-order design affects booking-event redundancy, order-tracking completeness, or alignment between ICU reservations and actual admissions. Related work on duplicate-order prevention [10], timestamp certification in emergency-response systems [11], and electronic health record (EHR)–driven error reduction [12] suggests that digital-order integrity is a critical but understudied component of high-acuity workflow reliability. However, these studies do not directly evaluate ED-initiated ICU admission pathways, leaving a gap in understanding how digital workflow redesign influences accuracy, traceability, and coordination in this specific transition.

This study evaluates how ED-initiated ICU admission workflows differed before and after deployment of a redesigned digital order system at a tertiary hospital. The updated system incorporates department-specific order separation, expanded timestamp capture, real-time documentation synchronization, and ICU bed-tracking integration. We hypothesized that these enhancements would improve the proportion of valid admission orders and reduce erroneous entries—including duplicates, lost orders, and tracking errors—thereby strengthening workflow accuracy and traceability in critical care transitions.

## Methods

We conducted a single-center retrospective, historically controlled, formative evaluation of the redesigned digital order system across a prespecified implementation window, using prospectively generated operational data from the ED and ICU.

### Study Design and Setting

The evaluation compared 2 prespecified 3-month periods at a tertiary medical center in Taiwan: from July to September 2023, when the traditional digital order system was in use, and from July to September 2024, after deployment of the updated system. The ED and ICU operate within a centralized digital infrastructure, with ICU bed reservations managed through an internal bed-coordination platform. Operational variables were defined before implementation, and all workflow events and timestamps were captured automatically and prospectively by the digital system during routine clinical operations.

Prior to the system redesign, ED-initiated ICU admission requests exhibited several operational gaps. Erroneous admission orders occurred in approximately 20% to 25% of booking events, duplicate orders in 5% to 10%, and lost or untracked orders in 2% to 4%. These discrepancies contributed to mismatches between ICU reservations and actual admissions, increased manual reconciliation workload, and reduced the reliability of downstream bed-management processes. These baseline characteristics motivated the development of a redesigned digital ordering system aimed at improving accuracy, traceability, and workflow efficiency.

This study represents an early evaluation of the updated system, consistent with the formative scope of this work. The selected 3-month postimplementation period reflects the earliest stable operational phase after deployment. Longer preintervention and postintervention periods will be analyzed once data consolidation is complete, and the refined system has reached a stable operational state suitable for longitudinal extraction.

### Intervention: Updated Digital Order System

In July 2024, the hospital implemented a modular, timestamp-driven digital order system designed to improve traceability, reduce documentation errors, and standardize ICU bed-reservation workflows. The updated system introduced structured order templates, automated timestamp capture at each booking step, department-specific separation of ED and non-ED orders, and a unified backend linking admission orders to their corresponding ICU reservation records. These refinements replaced the traditional 2023 workflow, which relied on free-text entries with minimal validation logic and lacked consistent mapping between booking orders and reservation outcomes.

In routine clinical operations, the updated system also provides real-time ICU bed-status displays and records booking-response actions; however, for this study, only the modules directly related to ED-initiated ICU booking orders and ICU reservation records were analyzed. These modules generate structured booking entries, enforce standardized timestamp capture, and maintain reservation-level identifiers used to evaluate order accuracy and reservation-to-admission alignment.

Basic operational information (ED volume, ICU census, and monthly ICU admissions originating from the ED) was examined to verify that no major workload shifts occurred between the 2 study periods. System-level data were obtained from the hospital’s integrated digital operational system, which combines the admission-order module and the ICU bed-management module within a unified backend. The admission-order component records all ED-initiated booking orders, including valid and erroneous orders and their associated error subtypes. The bed-management component captures reservation-level and admission-level information, including ICU bed reservations, ICU admissions, and final patient disposition. In this study, ICU bed reservations refer exclusively to reservations generated from ED-initiated ICU booking requests. Reservations originating from other hospital units were not included. Each reservation corresponds to a unique booking request recorded in the digital system, regardless of whether the patient was ultimately admitted to the ICU or to a general ward, transferred to another hospital, or discharged. This definition ensures alignment with the emergency-to-ICU workflow under evaluation and provides a consistent denominator for assessing admission-to-reservation alignment and reservation-level efficiency.

Two complementary datasets were extracted from the integrated operational system to support the analysis. The first was a visit-level input dataset, in which each ED encounter generated a row capturing the admission-order activity associated with that visit. This dataset reflected the clinician-entered inputs recorded during routine care.

The second was a booking-event output dataset, created by extracting all ED-initiated ICU booking events from the system’s backend. Each admission order corresponded to a booking event, and events were aggregated into weekly and monthly totals for statistical analysis. Because a single ICU reservation may be linked to more than one booking event (eg, a valid order plus a duplicate order), booking-event counts can exceed reservation counts. Erroneous events can contain more than one error subtype, so subtype totals can exceed the number of erroneous events.

Patient-level order activity was extracted from the integrated operational system for 6 monthly cohorts (July 2023, August 2023, September 2023, July 2024, August 2024, and September 2024). Each row represented a unique ED visit, identified by medical record number and ED arrival timestamp. For each visit, the dataset included (1) the total number of admission orders; (2) counts of erroneous orders, duplicate orders, lost orders, and tracking errors; and (3) the ED disposition code recorded at departure (0=death; 1=medical ICU; 2=cardiovascular ICU; 3=surgical ICU; 4=Tamsui surgical ICU; 5=Tamsui surgical neurological ICU; 6=general ward; 7=transfer to other hospitals; 8=discharged to home; 10=against-advice discharge; 11=catheterization laboratory; 12=operating room).

Manual recording of all booking-record entries was performed by a researcher who was blinded to the study design, study years, and the timing of the digital refinement. A second researcher, trained in structured spreadsheet-based data handling (including coding memos, item construction, and systematic checks for missing or inconsistent entries), identified error types by comparing the manually recorded dataset with the digitally extracted data using predefined rules, and a third researcher, experienced in spreadsheet-based data verification, independently validated all identified errors. No imputation or recoding was performed after manual review. Although manual classification introduces some potential for misclassification, the structured format of the digital booking record and the limited number of predefined error categories help mitigate this risk. These procedures were applied consistently across all months included in the analysis.

Erroneous orders were classified into duplicate, lost, and tracking-input errors. A subset of erroneous orders (n=22) had erroneous=1 but no subtype flag because the corresponding system-level reason codes were absent; these events were retained as unclassified erroneous orders to preserve the event-level denominator. No additional data processing was performed other than converting text-formatted numbers into numeric type for consistent handling across months. Timestamp discrepancies between clinician-visible times and backend logs reflect normal asynchronous processing and do not represent distinct error types; these artifacts became detectable only after timestamp refinement and are unrelated to the unclassified erroneous orders.

### Outcome Measures

Primary outcomes included the proportions of valid admission orders and erroneous orders. Secondary outcomes included the frequency of each error subtype and the admission-to-reservation ratio as a proxy for ICU bed allocation efficiency.

### Emergency-to-ICU Admission Order Metrics

#### Admission Order Validity and Error Classification

Admission order validity was assessed using ICU bed reservations as the denominator, reflecting the number of emergency-to-ICU reservations processed by each system. Orders were classified as valid or erroneous based on predefined criteria. Erroneous orders were further categorized into three subtypes: (1) duplicate–multiple orders for the same patient within a short interval; (2) lost–defined as orders not linked to any follow-up information (eg, reservation, discharges, or outcome documentation); and (3) tracking errors–defined as orders with inconsistent or missing timestamp sequences.

#### System Efficiency Metrics

System-level efficiency was evaluated by comparing ICU bed reservations and overall booking activity between the traditional (2023) and updated (2024) systems. The following metrics were used to evaluate system-level efficiency: (1) the admission-to-reservation ratio, defined as the number of ICU admissions divided by ICU reservations in each study period, reflecting reservation efficiency; (2) valid and erroneous order proportions, calculated using total booking events as denominators to characterize booking-event–level reliability; and (3) overall booking counts, reported descriptively to characterize system‑level demand and reservation activity. These efficiency metrics were evaluated using booking‑event data and ICU‑reservation information, with detailed findings presented in the “Results” section.

### Statistical Analysis

Weekly analytic units were defined by aggregating all ICU booking events within each calendar week. Weekly total events were calculated as the sum of order times for all encounters in that week, thereby capturing repeated and duplicate booking attempts for the same patient. Weekly counts of erroneous, duplicate, lost, and tracking-error orders were obtained by summing the corresponding fields across all booking events in that week. Weekly disposition counts were calculated as the total number of encounters in each category. Continuous weekly measures were summarized using the minimum, maximum, and median values.

Analyses were conducted using weekly and monthly aggregates derived from the booking-event output dataset, supplemented by visit-level characteristics from the input dataset. Booking-event variables—including counts of valid and erroneous admission orders, error-subtype frequencies, ICU bed reservations, and ICU admissions—were aggregated into weekly and monthly totals to evaluate order validity, error-domain patterns, and reservation-level efficiency. Visit-level variables were used to characterize the ED disposition patterns.

Comparative analyses of event-level outcomes were performed using Pearson chi-square tests for differences in proportions. Risk ratios (RRs) with 95% CIs and absolute differences in proportions were reported as conservative effect-size estimates for common outcomes. Admission-to-reservation ratios were analyzed as rates rather than proportions because the numerator (ICU admissions) and denominator (ICU reservations) represent different event sets. Rates were compared using Poisson regression with ICU reservations as the offset variable, whereas proportions—where the numerator and denominator refer to the same event set—were evaluated using Pearson chi-square tests. A forest plot was generated to visualize risk ratios with 95% CIs for admission-order outcomes associated with ICU requests originating from the ED. The plot provides a visual summary of effect sizes, while statistical inference in the manuscript is based on chi-square tests, risk ratios, and absolute differences.

All statistical tests were 2-sided with a significance threshold of α=.05. All analyses were conducted using R (version 4.3.1; R Foundation for Statistical Computing, Vienna, Austria).

### Ethical Considerations

This study used deidentified operational data extracted from hospital information systems. Institutional review board approval was obtained from the MacKay Memorial Hospital Institutional Review Board (MMH-IRB) under protocol number 26MMHIS082e. The study procedures met institutional criteria for minimal-risk operational research involving secondary analysis of deidentified data. No patient-identifiable information was accessed, and informed consent was waived. All procedures were conducted in accordance with relevant institutional and regional guidelines governing the use of deidentified operational data for quality improvement and system-evaluation research.

## Results

### Weekly Booking‑Event Patterns

Weekly booking-event volumes were broadly stable across the study period, with a median of 33 (range 18‐45) events per week in 2023 and 30 (range 6‐43) events per week in 2024 (Table 1). Each booking order—accurate or erroneous—corresponded to one booking event, and some patient reservations generated more than 1 booking order due to duplicate submissions, resulting in booking-event counts that exceeded reservation counts. Weekly erroneous events showed a downward shift after implementation of the refined system in 2024, decreasing from a median of 9 (range 2‐19) events per week in 2023 to 7 (range 0‐12) events per week in 2024. The sum of error-subtype counts frequently exceeded the number of erroneous events because a single erroneous booking event could carry more than one subtype. No unclassified erroneous events occurred in 2023, whereas granular timestamp logging in 2024 enabled detection of asynchronous processing delays, and erroneous events lacking subtype annotations (n=22) were categorized as unclassified. These weekly patterns provide background context, indicating that the observed increase in accurate-order rate after digital refinement reflects changes in system performance rather than differences in underlying booking volume or historical-control design.

**Table 1.** Weekly counts of total and erroneous events over the study period^a^.

Week	Total events	Accurate	Erroneous duplicate	Duplicate	Lost	Tracking	Unclassified
2023‑W26	20	11	9	10	4	0	0
2023‑W27	37	19	18	24	26	0	0
2023‑W28	30	15	15	12	23	0	0
2023‑W29	29	27	2	0	2	1	0
2023‑W30	30	22	8	4	6	0	0
2023‑W31	41	22	19	14	11	5	0
2023‑W32	39	24	15	14	14	0	0
2023‑W33	39	24	15	14	14	0	0
2023‑W34	24	19	5	0	5	1	0
2023‑W35	36	31	5	4	3	0	0
2023‑W36	34	25	9	0	5	4	0
2023‑W37	29	21	8	8	6	4	0
2023‑W38	45	30	15	14	14	0	0
2023‑W39	18	14	4	0	3	1	0
2024‑W27	20	20	0	0	0	0	0
2024‑W28	43	32	11	4	3	2	2
2024‑W29	35	29	6	0	3	0	3
2024‑W30	34	23	11	4	1	2	4
2024‑W31	35	26	9	6	0	1	2
2024‑W32	30	23	7	4	1	0	2
2024‑W33	24	18	6	2	2	0	2
2024‑W34	32	21	11	4	2	3	2
2024‑W35	27	23	4	2	0	0	2
2024‑W36	33	21	12	6	3	2	1
2024‑W37	29	21	8	2	2	3	1
2024‑W38	27	24	3	2	0	0	1
2024‑W39	25	15	10	2	8	0	0
2024‑W40	6	5	1	0	0	0	1

a Total events represent all admission-related digital booking records generated per ISO week. Each booking order—accurate or erroneous—corresponds to one booking event; therefore, total booking events equal the total number of booking orders. A single patient reservation may generate more than 1 booking order, so weekly booking-event counts can exceed reservation counts. Error-subtype totals may exceed erroneous-event totals because a single erroneous booking event can carry multiple subtype flags. No unclassified erroneous events occurred in 2023 because the traditional system lacked timestamp granularity. In 2024, granular timestamp logging made asynchronous processing delays detectable, and erroneous events without subtype annotations (n=22) were categorized as unclassified.

### Booking-Event–Level Transmission Performance

A total of 267 and 266 ICU admissions originating from the ED were processed during the traditional (2023) and updated (2024) digital ordering system periods, respectively (Table 2). Corresponding ICU bed reservations totaled 393 in 2023 and 343 in 2024. Overall ED-initiated ICU booking counts were 549 in 2023 and 399 in 2024, reflecting a reduction in redundant or unlinked booking activity under the updated system. Among 98 erroneous orders in 2024, a total of 76 had a recorded subtype (duplicate, lost, or tracking), while 22 were unclassified due to missing subtype annotation.

**Table 2.** Booking-event–level transmission performance under the traditional and updated digital ordering systems^a^.

Metric	Traditional system	Updated system
Total booking events, n	549	399
Valid admission orders, n (%)	297 (54.10)	301 (75.44)
Erroneous admission orders, n (%)	252 (45.90)	98 (24.56)
Duplicate orders, n (%)	110 (20.04)	38 (9.52)
Lost orders, n (%)	131 (23.87)	25 (6.27)
Tracking errors, n (%)	18 (3.28)	13 (3.26)

a This table summarizes all booking events generated during emergency department requests for ICU admission, including both valid and erroneous orders. Percentages use the total number of booking events as denominators (549 for the traditional system; 399 for the updated system). Error subtypes are not mutually exclusive and represent system-flagged issues within the digital transmission workflow. Reservation-level efficiency metrics are presented separately in Table 3.

### Admission Order Validity and Error Classification

Admission order validity improved following the system implementation (Figure 1, Table 3). Valid orders increased from 54.10% (297/549) in 2023 to 75.44% (301/399) in 2024, corresponding to a risk ratio of 1.39 and an absolute difference of 21.34% (*χ*_1_²=45.2; *P*<.001). Erroneous orders decreased from 45.90% (252/549) to 24.56% (98/399), with a risk ratio of 0.54 and an absolute difference of –21.34% (*χ*_1_²=49.1; *P*<.001).

**Figure 1.**
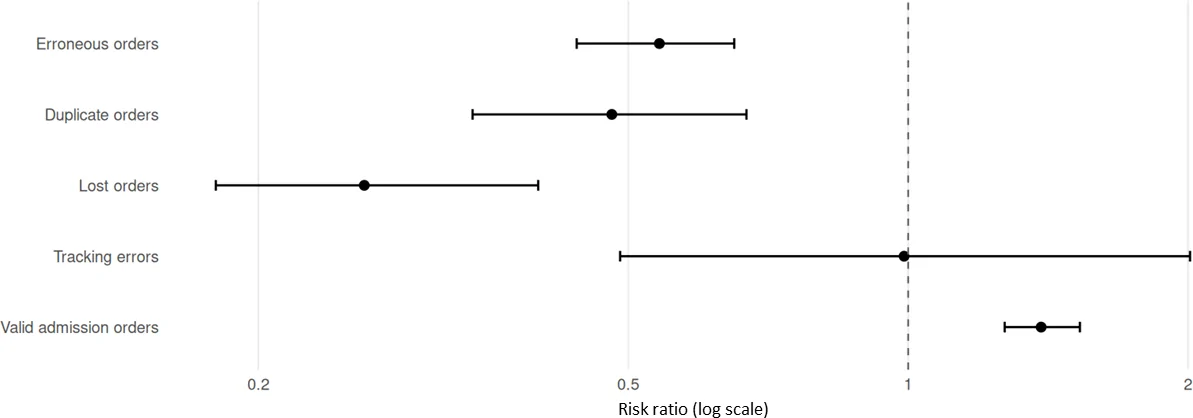
Risk-ratio forest plot comparing 2024 digital-refinement performance with the 2023 baseline. Risk ratios (RRs) and 95% CIs are shown for 5 outcomes—erroneous orders, duplicate orders, lost orders, tracking errors, and valid admission orders. RRs<1 indicate lower risk in 2024 relative to 2023. Digital refinement was associated with lower risks of erroneous, duplicate, and lost orders, with no change in tracking-error risk; valid-order rates increased.

**Table 3.** Admission-order validity and error outcomes by system type^a^.

Outcome	RR^b^	Absolute difference, %	*χ*² (*df*)^c^	*P* value
Valid admission orders	1.39	+21.34	45.2 (1)	<.001
Erroneous admission orders	0.54	−21.34	49.1 (1)	<.001
Duplicate orders	0.48	−10.52	19.4 (1)	<.001
Lost orders	0.26	−17.60	52.0 (1)	<.001
Tracking errors	0.99	−0.02	0.0003 (1)	.99

a This table summarizes booking-event–level comparative outcomes for all digital admission orders generated in response to emergency department requests for ICU admission. Odds ratios (ORs) with 95% CIs, risk ratios (RRs), absolute differences in proportions, and chi-square *P* values are used to compare the updated system with the traditional system. Percentages and raw counts for each outcome are reported in Table 2. Error subtypes are not mutually exclusive.

b ORs reflect logistic contrasts, while RRs and absolute differences provide conservative effect-size interpretations for common outcomes.

c Chi-square tests compare proportions between systems.

Among the error subtypes, duplicate orders declined from 20.04% to 9.52%, with a risk ratio of 0.48 and an absolute difference of –10.52% (*χ*_1_²=19.4; *P*<.001); lost orders decreased from 23.87% to 6.27%, with a risk ratio of 0.26 and an absolute difference of –17.60% (*χ*_1_²=52.0; *P*<.001); and tracking errors remained stable at 3.28% in 2023 and 3.26% in 2024, with a risk ratio of 0.99 and an absolute difference of –0.02% (*χ*_1_²=0.0003; *P*=.99).

These findings are summarized in Table 3 and visually synthesized in Figure 1, which displays risk ratios with corresponding CIs for each outcome. The plot highlights substantial reductions in lost and duplicate orders, while tracking errors remain centered near the null line.

### System Efficiency Metrics

Admission‑to‑reservation ratios differed between years, rising from 0.68 to 0.78, with a corresponding rate ratio of 0.88 (95% CI 0.77-0.99; *P*=.03), consistent with more efficient reservation patterns under the updated system (Table 4).

**Table 4.** ICU reservation efficiency and clinical-volume metrics^a^.

Metric	Traditional system	Updated system	Rate ratio (95% CI)	*P* value
ICU^b^ bed reservations	393	343	—^c^	—
ICU admissions from ED^d^	267	266	—	—
ICU admission-to-reservation ratio^e^	0.68	0.78	0.88 (0.77‐0.99)	.03

a This table summarizes reservation-level operational metrics, including ICU bed reservations, ICU admissions originating from the emergency department, and the admission-to-reservation rate. The rate reflects reservation efficiency and was compared across systems using a Poisson regression model with ICU reservations as the exposure (offset). Reservation-level metrics are conceptually distinct from booking-event–level reliability outcomes presented in Table 2.

b ICU: intensive care unit.

c Not available.

d ED: emergency department.

e The rate is calculated as ICU admissions divided by ICU bed reservations; lower rates indicate greater reservation efficiency. Statistical comparison was performed using Poisson regression with ICU reservations as the exposure (offset).

### Summary

The updated digital order system was associated with statistically significant improvements in admission-order validity and reductions in erroneous, duplicate, and lost orders. System-level efficiency also improved, as reflected by fewer ICU reservations per admission and reduced booking redundancy. The forest plot (Figure 1) provides a visual summary of these differences using risk ratios with 95% CIs, complementing the effect-size estimates reported in Table 3. Tracking-error rates did not differ meaningfully between systems, indicating an area for further refinement.

## Discussion

### Principal Findings

Within this evaluation, the updated digital order system relied primarily on routinely captured operational data, with no observable increase in operational errors during ED-ICU transitions. The platform recorded workflow events with consistent timeliness and accuracy, and the observed improvements in admission-order validity, reservation stability, and error frequencies coincided with reduced reliance on manual recording rather than added documentation burden. These patterns suggest a shift toward a digital-first workflow, in which structured, timestamp-driven event capture is associated with more consistent coordination and closer alignment between admission intent and ICU resource allocation, without implying a demonstrated capability or a causal effect.

The updated system was associated with improvements in emergency-to-ICU admission performance, reflected in higher order validity, fewer duplicate and lost orders, and more consistent reservation efficiency. These observed patterns align with the value of structured, timestamp-driven digital infrastructures in supporting reduced operational variability and more predictable ICU resource coordination, without implying demonstrated effect. The findings are consistent with literature on structured electronic order systems, digital-workflow standardization, and timestamp integrity, all of which emphasize the importance of consistent data capture and traceable event streams for reliable clinical operations. The observed alignment between admission orders and reservation records further reflects the relevance of emergency-to-ICU coordination for downstream resource allocation. As hospitals continue modernizing their digital ecosystems, these results suggest that system-level digital-workflow refinements may coincide with measurable operational gains and help establish a foundation for broader workflow optimization in future evaluations.

### Interpretation of Key Findings

The most substantial differences were observed in the lower frequencies of duplicate and lost orders, both of which represent critical vulnerabilities in emergency workflows. Duplicate orders often arise from fragmented communication or repeated attempts to secure ICU beds, whereas lost orders indicate failures in linking admission intent to downstream actions such as reservations or discharge documentation. Automated timestamp capture and structured order templates coincided with lower frequencies of these inefficiencies, describing short-term operational patterns associated with the updated system. These differences were reflected in a risk ratio of 0.54 and an absolute reduction of 21.3 percentage points in erroneous orders, describing a lower error frequency during the updated-system period. This interpretation is consistent with extensions of the DeLone and McLean IS Success Model, which suggest that improvements in EHR system quality, information quality, and service quality may contribute to fewer reported errors and disruptions in clinical workflows [12-14].

In contrast, tracking errors did not differ significantly between systems. This suggests that while order validation has improved, challenges remain in ensuring complete timestamp integrity. In our setting, most tracking errors occurred when patients were admitted to the ICU of the branch hospital, but the originating booking record did not receive the corresponding admission label, resulting in mislabeled or unlabeled events. This pattern indicates that tracking-related discrepancies may arise from cross-site data-flow gaps rather than from the order-entry interface itself, and further refinement of audit trails and interhospital synchronization will be required to address this residual source of error.

### System Efficiency and Operational Implications

The lower admission-to-reservation ratio, decreasing from 1.47 to 1.29 during the updated-system period, coincided with more consistent alignment between admission orders and ICU bed reservations. Fewer redundant bookings were observed, indicating reduced coordination load and closer alignment between admission intent and reservation actions.

### Comparison With Prior Literature

Prior evaluations of digital order systems have consistently shown that structured electronic workflows reduce documentation errors, improve traceability, and enhance coordination across clinical units. Studies examining Internet of Things–enabled and interoperable digital infrastructures report that improved system integration enhances workflow efficiency and data integrity [8]. Broader analyses of digital emergency-response systems similarly highlight the operational relevance of timestamp-driven coordination and real-time information flow [9]. Evidence from EHR-based decision-support interventions further demonstrates that passive, inline prompts can reduce unintentional duplicate orders, supporting our observed reductions in duplicate and lost orders [10]. These findings collectively reinforce the importance of structured, timestamp-driven digital pathways in strengthening reliability in high-acuity admission workflows.

Our findings describe how system-level digital workflow refinements were associated with differences in ICU admission processes, a domain where timeliness and accuracy are critical. The lower frequencies of duplicate and lost orders are consistent with prior reports of enhanced order traceability following digital interventions, while the persistence of tracking-related discrepancies highlights the need for continued optimization of cross-site data flows and audit-trail synchronization.

Research on ED-ICU digital handover processes similarly emphasizes the importance of accurate, timestamped information transfer for timely ICU placement and bed-management efficiency. Prior work has shown that mismatches between admission intent and reservation records contribute to operational uncertainty and delayed ICU allocation. The more consistent alignment between ICU reservations and actual admissions in our study aligns with prior reports and suggests that lower upstream order variability may coincide with more stable downstream capacity-management patterns.

Most existing literature focuses on patient-level outcomes, clinician-reported usability, or broad EHR adoption metrics. In contrast, our study evaluates booking-event–level reliability, an operational signal rarely examined in digital-health research. By quantifying error-subtype patterns, duplicate-order frequency, and reservation alignment, this work contributes a more granular perspective on how digital redesign affects the integrity of operational data streams. Prior studies have called for more detailed operational metrics to complement traditional clinical outcomes; our findings provide empirical support for this approach by demonstrating early stabilization of event-stream reliability following system implementation.

Finally, while many digital-intervention studies rely on extended pre- or postintervention periods or full interrupted time-series designs, few have characterized early-stage system performance immediately after hospital-wide deployment. This study adds to the evidence base by documenting short-term patterns in order validity and reservation efficiency during the initial stabilization period. These results highlight the value of early operational monitoring and underscore the potential for rapid gains following targeted digital-workflow redesign. This interpretation is further supported by evidence showing that improvements in EHR system quality, information quality, and service quality are associated with measurable reductions in medical error, as demonstrated in a recent extension of the DeLone and McLean IS Success Model [12-14].

### Clinical and Informatics Significance

From a clinical perspective, lower frequencies of erroneous orders are associated with more reliable documentation of ICU admission intent. Differences in order validity and the lower frequencies of duplicate and lost orders describe patterns consistent with safer and more consistent transitions of care. From an informatics standpoint, the evaluation characterizes how modular system design and timestamp-driven logic were associated with short-term operational patterns during the updated-system period. These insights are particularly relevant for hospitals seeking to optimize digital workflows in high-acuity environments [11], where timestamp integrity and structured digital pathways are essential for reliable coordination.

More broadly, the findings describe how structured, event-driven digital workflows were associated with more consistent patterns in high-acuity transitions, including lower operational variability and closer alignment between admission intent and ICU resource allocation. The results also characterize how standardized order structures and consistent event capture coincide with more predictable, traceable, and coordinated care pathways across clinical units.

### Implications for Practice

The findings describe how digital platforms that capture operational events during execution were associated with reduced reliance on manual documentation in high-acuity settings. In this evaluation, timely and accurate system-generated records coincided with sustained workflow coordination without additional manual effort, characterizing how ED and ICU teams operated within a streamlined, digital-first process during the period with the updated-system period.

### Limitations

Although operational workload indicators—ED volume, ICU census, and ED-origin ICU admissions—were stable across periods, unmeasured institutional factors may still influence before-and-after comparisons. This evaluation was conducted at a single tertiary medical center, which may limit generalizability to institutions with different digital infrastructures, workflow structures, or ICU admission policies. The study focused on operational workflow metrics rather than patient-level outcomes, and qualitative assessments of staff experience or coordination burden were not included.

Tracking-related errors did not decrease during the study period, indicating that system-level refinements may not uniformly affect all error categories. This pattern suggests that certain error types may be more sensitive to workflow redesign than others and highlights the need for continued refinement of tracking logic and event-linkage mechanisms.

The analysis compared 2 predefined 3-month periods using a historical-control design. Although identical calendar months were selected to reduce seasonal effects, the approach remains susceptible to history bias from unmeasured external factors such as staffing patterns, ICU occupancy, or concurrent institutional initiatives. A continuous time series suitable for segmented regression was not feasible because the 2023 and 2024 cohorts are separated by a 9-month gap, preventing construction of an uninterrupted longitudinal sequence. Accordingly, the findings should be interpreted as formative, reflecting early-stage evaluation rather than causal inference. Longer observation windows will be examined once data consolidation is complete and the refined system reaches a stable longitudinal state suitable for extended analysis.

### Future Work

Future work should examine how the transition to a digital-first workflow influences documentation load and coordination effort in high-acuity settings. As the updated platform reduced reliance on manual recording by capturing operational events automatically during execution, a dedicated evaluation of workload diminution—using time-motion analysis, interaction-log metrics, or mixed-methods assessment—would clarify the extent to which digital event capture decreases cognitive and documentation burden for ED and ICU teams. Such analyses would complement the present findings by quantifying the operational impact of digital recording on staff effort and workflow sustainability.

### Conclusion

This study describes how the updated digital order system was associated with more accurate, timely, and aligned ED-ICU transitions, alongside lower reliance on manual documentation. In this evaluation, routinely captured operational data coincided with sustained real-time coordination, characterizing a shift toward a digital-first workflow in which automated event capture supported the timeliness and accuracy required for high-acuity processes. The short-term differences observed across order validity, error frequencies, and reservation efficiency describe patterns consistent with the role of structured, timestamp-driven infrastructures in supporting reliability and resource alignment in critical-care admissions. As hospitals continue to advance their digital ecosystems, these findings illustrate how targeted system-level redesign may coincide with measurable operational gains and help establish a foundation for broader digital-workflow optimization.

## Abbreviations

ED: emergency department
EHR: electronic health record
ICU: intensive care unit
MMH-IRB: MacKay Memorial Hospital Institutional Review Board
RR: risk ratio
